# Simulated responses of soil organic carbon stock to tillage management scenarios in the Northwest Great Plains

**DOI:** 10.1186/1750-0680-2-7

**Published:** 2007-07-24

**Authors:** Zhengxi Tan, Shuguang Liu, Zhengpeng Li, Thomas R Loveland

**Affiliations:** 1 SAIC, contractor to US Geological Survey (USGS) Center for Earth Resources Observation and Science, Sioux Falls, SD 57198, USA; 2 Geographic Information Science Center of Excellence, South Dakota State University, Brookings, SD 57007, USA; 3 US Geological Survey (USGS) Center for Earth Resources Observation and Science, Sioux Falls, SD 57198, USA

## Abstract

**Background:**

Tillage practices greatly affect carbon (C) stocks in agricultural soils. Quantification of the impacts of tillage on C stocks at a regional scale has been challenging because of the spatial heterogeneity of soil, climate, and management conditions. We evaluated the effects of tillage management on the dynamics of soil organic carbon (SOC) in croplands of the Northwest Great Plains ecoregion of the United States using the General Ensemble biogeochemical Modeling System (GEMS). Tillage management scenarios included actual tillage management (ATM), conventional tillage (CT), and no-till (NT).

**Results:**

Model simulations show that the average amount of C (kg C ha^-1^yr^-1^) released from croplands between 1972 and 2000 was 246 with ATM, 261 with CT, and 210 with NT. The reduction in the rate of C emissions with conversion of CT to NT at the ecoregion scale is much smaller than those reported at plot scale and simulated for other regions. Results indicate that the response of SOC to tillage practices depends significantly on baseline SOC levels: the conversion of CT to NT had less influence on SOC stocks in soils having lower baseline SOC levels but would lead to higher potentials to mitigate C release from soils having higher baseline SOC levels.

**Conclusion:**

For assessing the potential of agricultural soils to mitigate C emissions with conservation tillage practices, it is critical to consider both the crop rotations being used at a local scale and the composition of all cropping systems at a regional scale.

## Background

Many studies have identified the potential of soils cultivated with conservation practices (e.g., no-till) to sequester large amounts of carbon (C) [1,2]. It is estimated that conservation tillage practices across the United States may drive large-scale sequestration on the order of 24–40 Tg C yr^-1 ^(Tg: teragram; 1 Tg = 10^12 ^g), and that additional C sequestration of 25–63 Tg C yr^-1 ^can be achieved through other modifications to traditional agricultural practices [3]. In regard to the C credit scenario established by the Kyoto Protocol, it is widely suggested that conversion of conventional tillage (CT) to no-till (NT) can help to support the profitability of C credits for farmers. The uncertainties of these sequestration scenarios, however, depend on soil organic carbon (SOC) monitoring and/or models [2].

Recently, eddy-covariance measurements have been used to evaluate the contribution of NT practice to C dynamics in corn (*Zea mays *L.) and soybean (*Glycine max *(L.) Merr.) rotation ecosystems at regional and national scales [1,2]. However, the relationships between net ecosystem exchange (NEE) and either terrestrial C storage or actual SOC stocks are still poorly understood, owing to the uncertainty of the redistribution of biomass in farming products beyond a given C accounting region. For example, based on the assessment of NEE over six years, *Hollinger et al*. [2] estimated C stocks for corn/soybean rotation ecosystems in the North Central Region of the United States, and observed a C sink under NT at the local scale which, however, is not necessarily true on a regional scale. They attributed this discrepancy to regional consumption of grain combined with C emissions associated with agricultural practices.

Temporal variability in SOC stock is indicative of the response of ecosystems to changes in climate, land use/land cover, and land management. Because the dynamics of SOC directly impact the availability of nutrients and moisture to all kinds of living organisms, changes in SOC stock can transform the structure and functions of ecosystems, and may also result in ecosystem feedbacks on climate [4]. The General Ensemble biogeochemical Modeling System (GEMS) [5] has been used by *Tan et al*. [6] to simulate the terrestrial C dynamics in the Northwest Great Plains between 1972 and 2000. Results show that C sources of croplands and the SOC balances across the ecoregion depend on the proportion of cropped area to grassland. This study, however, did not take into account the contribution of land management to SOC dynamics.

How well we predict future atmospheric CO_2 _dynamics and their response to anthropogenic CO_2 _emissions depends on our understanding of the extent to which the rising atmospheric CO_2 _concentration can be offset by terrestrial ecosystems through conservation agricultural practices. Therefore, we need to evaluate the impacts of change trends in land use/land cover and land management on terrestrial C source-sink relationships associated with specific management practices.

Sequestering C in cultivated soils managed with NT is being advocated as a way to assist in meeting the demands of an international C credit system [1]. The potential of cropland with such conservation management to mitigate CO_2 _emissions from mixed grass-crop ecosystems at a regional scale needs to be evaluated. In this study, we simulated SOC dynamics within the top 20 cm of soil in the cropped areas of the Northwest Great Plains between 1972 and 2000 with three management scenarios: actual residue and tillage management (ATM), all cropland managed with CT, and all cropland managed with NT.

## Results

### Croplands and tillage management history

Major historical changes in land use/land cover within the ecoregion were directly related to conversions between cropland and grassland. In 1972 the average percentages of cropland and grassland were 17% and 75%, respectively, and changed to 15% and 77%, respectively, in 2000. For the study area, the average percentage of cropland from 1972 to 2000 was 41%, 21%, 9%, and 5% in North Dakota, Montana, South Dakota, and Wyoming, respectively.

**Figure 1:**
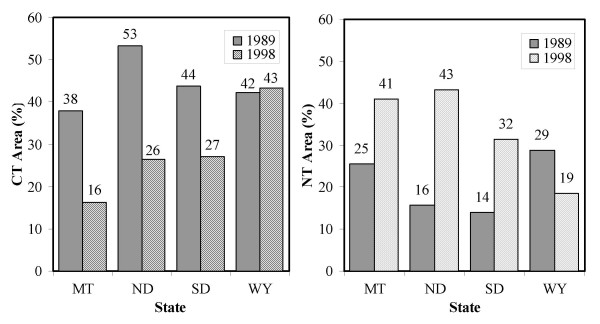
Changes in conventional tillage (CT) and no-till (NT) areas for each state between 1989 and 1998 in the Northwest Great Plains ecoregion.

Average cropped area between 1989 and 1998 was about 4.06 million ha within the ecoregion. As indicated in Figure 1, the CT area was 45% in 1989 and decreased to 24% in 1998. During the same period, the area of NT increased from 19% to 38%, and the reduced-tillage (RT) area changed slightly from 36% to 38%. But the change rate of each tillage-managed area varied from state to state (see Figure 1). For example, from 1989 to 1998, the NT area increased by 27% in North Dakota whereas it declined by 10% in Wyoming.

### Cropping systems and associations with tillage management

During the 1990s, there was little variation in the areal proportions of major cropping systems in the ecoregion, which consisted of 67% small grain crops (predominated by wheat), 12% corn, 8% soybean, and 14% others (Table 1). The areal proportion of each cropping system managed with NT, RT, and CT was 30%, 35%, and 35%, respectively, and not significantly different over time despite large differences among the four states.

**Table 1:** Percentage of individual cropping systems in the total planted area under each tillage management at the ecoregion scale.

Cropping system	NT	RT	CT	Total
Corn	4.2	3.7	3.6	11.5
Small grain	20.4	24.8	21.4	66.6
Soybean	2.9	2.4	3.1	8.4
Other	2.7	4.5	6.9	14.0
Total planted^a^	29.9	35.2	34.9	100.0

NT: no-till; RT: reduced tillage; CT: conventional tillage.

^a ^excluding fallow area.

### Changes in SOC pools with tillage management scenarios

For sample blocks with cropped area percentage greater than 10%, total SOC stock within the top 20 cm depth of soil generally tended to decrease with cultivation time, but the reduction in SOC stock was smaller under NT (210 ± 33 kg C ha^-1^yr^-1^) than under both CT (261 ± 36 kg C ha^-1^yr^-1^) and ATM (246 ± 38 kg C ha^-1^yr^-1^). The average reduction in the rate of C release with conversion of CT to NT was about 51 kg C ha^-1^yr^-1 ^during the study period. No significant difference between ATM and CT was observed. The reduction in the rate of C release, however, was correlated with the baseline SOC levels. For example, sample block 04 in North Dakota had a high baseline SOC stock (66 Mg C ha^-1^) and was simulated to have a reduction in the rate of C release at 104 kg C ha^-1^yr^-1 ^with NT compared with CT. In contrast, sample block 02 in Montana had a low baseline SOC stock (34.5 Mg C ha^-1^) and was simulated to show a reduction in the rate of C emission of 56 kg C ha^-1^yr^-1 ^with NT in comparison with CT. Simulation results also demonstrate that, by the year 2000, sample block 04 remained a C source at the rate of 330 kg C ha^-1^yr^-1^, whereas sample block 02 turned into a C sink at the rate of 9 kg C ha^-1^yr^-1^.

**Figure 2:**
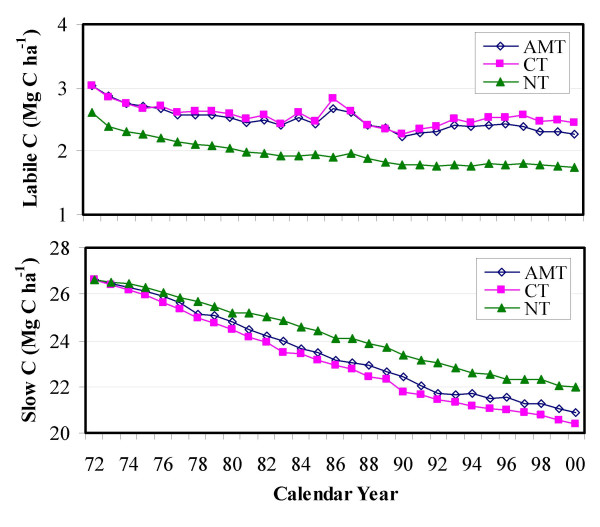
Temporal trends in soil labile C and slow C pools in association with conventional tillage (CT), no-till (NT), and actual tillage management (ATM) within sample blocks where cropped area was greater than 10% of the block area in the Northwest Great Plains ecoregion.

Simulation results indicate that the changes in total SOC stock with conversion of CT to NT were predominantly a result of changes in both the labile and slow C pools. Figure 2 shows that there was a relatively consistent reduction (about 0.7 Mg C ha^-1^) in the labile SOC pool under NT in comparison with CT (and ATM); whereas the slow pool increased by 1,6 Mg C ha^-1 ^by the year 2000. In other words, the conversion of CT to NT would reduce the slow C emissions at the rate of 57 kg C ha^-1^yr^-1 ^in the study area.

### Change rate of SOC pools in relation to baseline SOC levels

**Figure 3:**
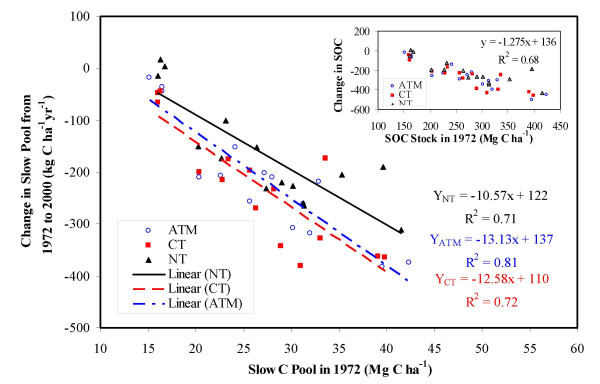
Magnitude of the change in the slow C pool in the top 20 cm depth of soil from 1972 to 2000 for three tillage scenarios in relation to the baseline slow pool in 1972 for the sample blocks in which the cropped area percentage was greater than 10%. The inset graph illustrates the relation of the change in total SOC stock to the baseline.  (ATM: actual tillage management; CT: conventional tillage; NT: no-till)

**Figure 4:**
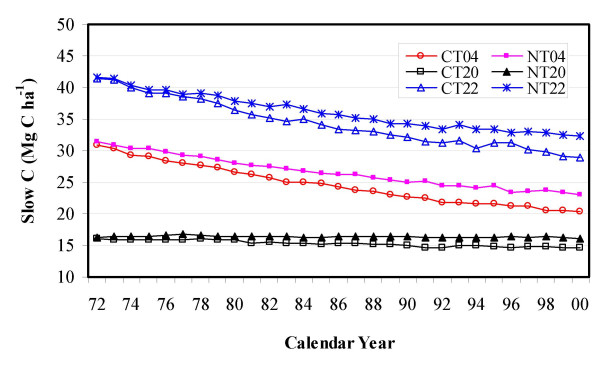
Temporal trends in slow SOC pools under CT and NT in association with various baseline SOC levels in 1972 at the sample block scale within the Northwest Great Plains ecoregion (The numbers 04, 20, and 22 refer to the sample block IDs. These three blocks are located in North Dakota, Montana, and North Dakota, respectively).

The data in Figure 3 indicate that crop production, regardless of tillage practices, tends to remove SOC from the top soil layer, even though there is less loss under NT than under CT. Responses of total SOC stock (especially of the slow C pool) to tillage management, however, depend significantly on baseline SOC levels. As illustrated in Figure 4, soils with higher baseline SOC content tend to lose more C following crop cultivation. Conversely, the conversion of CT to NT has less influence on SOC stocks for soils having lower baseline SOC levels, but would lead to higher potentials to mitigate the C release from soils having higher baseline SOC content. This is in agreement with the conclusion of *Tan et al*. [10] for the east central United States.

## Discussion

Our results indicate that the reduction in the rate of C emissions with conversion of CT to NT at the ecoregion scale is much smaller than those reported at the plot scale, and also much smaller than simulated values for other regions. For example, based on field-scale measurements of C flux between the atmosphere and the corn/soybean ecosystem in the United States for the years 1997–2002, *Bernacchi et al*. [1] demonstrate that current corn/soybean agricultural practices release more C than is removed from the atmosphere with 10% of the cropland being in continuous NT agriculture, but that this ecosystem can become a C sink estimated at a rate of 300 kg C ha^-1 ^yr^-1 ^with NT management implemented over a larger area. In fact, the responses of the SOC stock in the upper soil layer to tillage practices not only demonstrate considerable spatial variation, but also depend on cropping systems at local scales [11], and are even influenced by sampling protocols [12].

Many studies have shown that NT does not necessarily lead to C sequestration within the upper 20 cm depth of soil; NT can result in either C sources or sinks, depending on cropping systems [13,14]. Although relatively high reduction rates of C release have been reported for continuous corn and corn/soybean cropping systems, other cropping systems such as continuous soybean, cotton production, and wheat/summer fallow rotation are usually reported as C sources [13,15,16]. As indicated by the data presented in Table 1, for either the complete CT or the complete NT scenario in this study, two-thirds of the planted area was in small grain production dominated by wheat, with only about 12% for corn and 8% for soybeans. The management data (named as CTIC data afterwards) derived from the Conservation Technology Information Center [17] show that the fallow area was equivalent to 18% of the total planted area. This composition of cropping systems would determine the extent to which the SOC stock had been influenced by tillage practices during the study period.

*Huggins et al*. [11] assessed the effects of crop sequence and tillage on SOC stocks using the natural ^13^C abundance of corn (*Zea mays L.*) and soybeans (*Glycine max (L.)*, *Merr.*). Soil samples were collected after 14 years under each treatment for SOC quantification. They observed the influence of crop sequence on SOC (0 – 45 cm depth) that occurred when tillage was reduced with chisel plow and NT. Results show that there was 15% more SOC in continuous corn than in continuous soybean, but all tillage treatments within continuous soybean systems showed little influence on SOC. *Peterson et al*. [18] conducted CT-NT paired experiments on wheat-dominated cropping systems in Mandan, North Dakota and found a small annual increase rate (about 0.25%) of the SOC pool with NT, which is very close to our result averaged at the ecoregion scale.

*Halvorson et al*. [16] documented that the SOC stock did not increase during 12 years under a spring wheat/fallow system with NT in North Dakota. Compared with CT, the NT tended to lose more SOC at a rate of 50 kg ha^-1^yr^-1 ^in the upper 15 cm depth under a spring wheat/fallow system, which seems to support the conclusion that soil C in the surface layer can be quickly lost to the atmosphere by increasing summer fallow practice [19]. A similar result was also reported by *Campbell et al*. [20] in Canada. *Halvorson et al*. [16] suggested that conversion from crop/fallow to more intensive cropping systems with NT is needed in order to have a positive impact on reducing CO_2 _emissions from croplands in the Northern Great Plains ecoregion.

Based on metadata analyses with a large number of point observations from published works, *Manley et al*. [21] concluded that NT is less effective for sequestering C on the Prairies than in other regions (e.g., in the southern United States and the Corn Belt), and also less effective with wheat than with other crops. Generally, NT for either a continuous corn system or a corn/soybean rotation system leads to C sinks and more net C gain comes with a longer duration of NT [2], which, however, depends on baseline SOC contents [10] and tends to level off as the soil becomes saturated [22]. Unfortunately, this study could not define the saturation level for the whole study area because we don't have enough long-term management data and necessary field observations to drive model simulations. Furthermore, the saturation level varies greatly not only with specific soil but also with other many factors.

Using historical county-level land use data for the 19th and 20th centuries to drive an ecosystem model, *Parton et al*. [15] conducted four case studies within the Great Plains of the United States that were used to represent different agro-ecosystems. Model results show that cultivation of grassland results in large losses of SOC and an increase in soil nitrogen mineralization for the first 20 to 30 years of cultivation, followed by small SOC loss and nitrogen mineralization after 50 years cultivation. Their simulation results also indicate that the irrigated cotton production would lead to a net C source whereas the irrigated corn and alfalfa cropping systems would result in a C sink in the central and northern Great Plains.

Our estimate of the limited reduction in the rate of SOC release with conservation tillage management across the Northwest Great Plains could be attributed to the wheat/fallow-dominated crop rotation and the composition of all cropping systems being practiced in this ecoregion.

## Conclusion

Our simulated reduction in the rate of C release with conversion of CT to NT in the agricultural soils across the Northwest Great Plains ecoregion between 1972 and 2000 is much smaller than those reported from plot scale studies and also smaller than simulated values for other regions in general. However, similar estimates are reported by other investigators for the crop rotations and composition of cropping systems similar to those in our study area. The changes in total SOC stock were predominantly a result of the dynamics of the slow C pool at the study's time span. We suggest that the responses of total SOC to tillage management scenarios depend significantly on the baseline SOC level. Soils with higher SOC levels tend to have higher potentials to reduce C emissions with conservation tillage practices, but the dominance of the wheat/fallow crop rotation and the composition of all cropping systems could be the primary cause for the limited efficiency of NT for mitigating C emissions as simulated for the Northwest Great Plains ecoregion.

## Methods

### Study area

**Figure 5:**
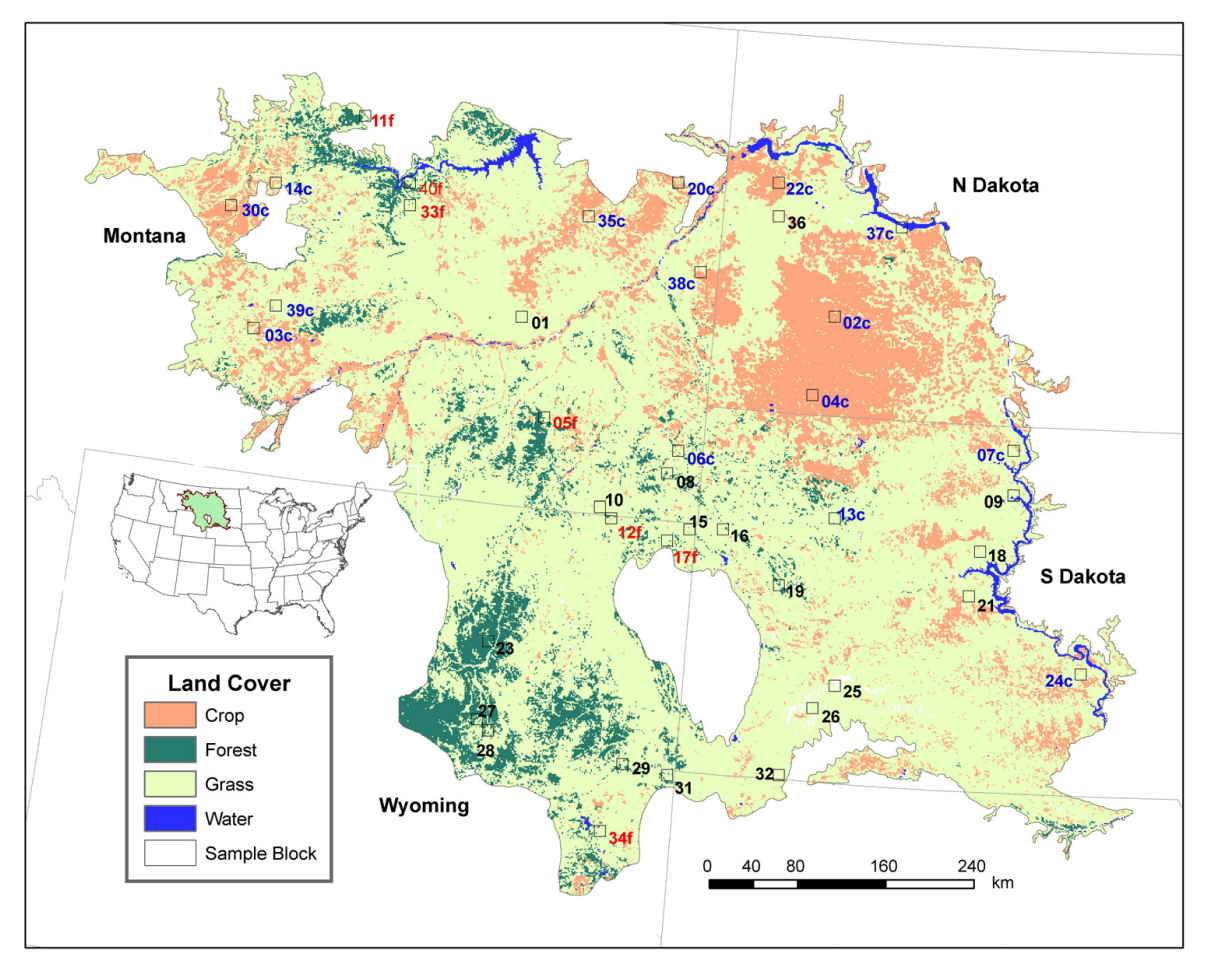
Study area and locations of sample blocks (After *Tan et al*. [7]).

Our study area is the Northwest Great Plains ecoregion, with a land area of 338,718 km^2 ^(Figure 5). The average annual precipitation from 1972 to 2000 was 400 mm (wetter in the eastern portion of the ecoregion), and the average annual temperature was 7.2°C (warmer in the southern portion of the ecoregion) [6]. The mean annual temperature was 0.67°C higher between 1986 and 2000 than between 1972 and 1985.

As illustrated in Figure 5, the land cover in the ecoregion consisted of 75% mixed grasses, 17% cropland, and 8% other land covers, on average, from 1972 to 2000 [6]. Agriculture, however, has been the primary land use transforming this grassland-dominated ecosystem. Cumulative change in land cover during the period accounted for about 10% of all land area, but most of these changes were directly related to conversions between cropland and grassland that resulted from the implementation of the Conservation Reserve Program (CRP) [6]. Cropped areas were mainly located on level ground where soils were fertile and devoted to row crops, small grains, and fallow. Major crop types included spring wheat (*Triticum aestivum*), corn (*Zea mays *L.), soybean (*Glycine max *(L.) Merr.), and alfalfa (*Medicago sativa*). The proportion of all cropland to the total land area varied from state to state, ranging from 5% in Wyoming to 46% in North Dakota.

### Sampling framework

The sampling protocol proposed by *Loveland et al*. [8] was introduced to identify spatial variation of land use/land cover change in the conterminous United States using Omernik's 84 Level III Ecoregions [9] as the sampling framework. For the Northwest Great Plains ecoregion, forty sample blocks of 10 km × 10 km each were randomly selected (Figure 5) to identify changes with a precision of 1% at an 85% confidence level [8]. Changes were detected based on five dates (1973, 1980, 1986, 1992, and 2000) of Landsat Multispectral Scanner (MSS) and Thematic Mapper (TM) imagery data that were analyzed at a cell size of 60 m × 60 m for MSS images and 30 m × 30 m for TM images.

### Modeling system

The General Ensemble biogeochemical Modeling System (GEMS) [5] was used to simulate soil C dynamics in this study. GEMS is a modeling system developed for a better integration of spatially explicit time-series land use and land cover change data with well-established ecosystem biogeochemical models (e.g., CENTURY). GEMS has been used to simulate C dynamics in vegetation and soil for diverse ecosystems, especially in the northwest Great Plains [6,7]. As described by *Liu et al*. [5] and Tan et al. [7], GEMS consists of three major components: single or multiple encapsulated ecosystem biogeochemical models, an automated stochastic parameterization system (AMPS), and an input/output processor (IOP). AMPS includes two major interdependent parts: the data search and retrieval algorithms and the data processing mechanisms. The first part searches for and retrieves relevant information from various databases according to the keys provided by a joint frequency distribution (JFD) table. The data processing mechanisms downscale the aggregated information at the map-unit level to the field scale using a Monte Carlo approach. Once the data are assimilated, they are injected into the modeling processes through the IOP which updates the default input files with the assimilated data. Values of selected output variables are also written by the IOP to a set of output files after each model execution. The JFD grids are first created from soil maps, a time-series of land cover images, and climate themes at a cell size of 60 m × 60 m. The CENTURY model [23] was selected as the underlying ecosystem biogeochemical model in GEMS for this study because it has solid modules for simulating C dynamics at the ecosystem level and has been widely applied to various ecosystems worldwide.

### Input data for model

The spatial simulation unit of GEMS is a JFD case. A JFD case contains single or multiple, homogeneous, connected or isolated land pixels that represent a unique combination of values from the Geographic Information System (GIS) layers [5]. The data for model input primarily consisted of climatic regimes, land use/land cover change, soil inventory, management data, nitrogen (N) deposition map, and administrative districts. Land use/land cover data were described above. Climatic data consisted of annual precipitation and maximum and minimum temperature records from 1973 through 2000, which were converted to 30 m pixel GIS layers from the CRU TS 2.0 datasets [24]. Soil characteristics within each sample block were taken from the US State Soil Geographic Data Base (STATSGO) [25] for initializing the soil components of GEMS. Tillage management data were derived from the CTIC [17] and would be discussed in detail below.

GEMS automates the processes of downscaling forest ages from the USDA Forest Inventory and Analysis data (FIA), crop compositions from the Agricultural Census, grass cover distribution and temporal changes from the remotely sensed imagery interpretation. A Monte Carlo method was used to assign each JFD a set of specific soil property values such as layer depth, soil organic matter content, soil water holding capacity, and clay and sand percentages. Based on the definitions set by CENTURY, we partitioned the SOC stock into different pools at the beginning of each simulation using a retrospective SOC initialization algorithm: the slow SOC pool was defined from the NPP for each land cover type and soil inventory data; the difference between the total SOC and the slow pool was then used to initialize the passive SOC pool; and the active SOC pool was set at about 2% of the total SOC storage [7].

### Ensemble simulations

GEMS generates site-level inputs with a Monte Carlo approach from regional data sets. Any single simulation of a JFD case is unique combination of randomly picked forest age, crop species, and soil properties from regional-level datasets, so that the output of a single simulation run of a JFD might be biased. Therefore, ensemble simulations of each JFD were executed to incorporate the variability of inputs and to average uncertainties of simulation results. In general, the averages of ensemble simulations become more stable when increasing the the times of run. We made 20 repeat runs for each JFD case in this study, which reduced the relative error to about 2%. The averaged JFD output from the 20 runs was then aggregated at sample block scale, and the simulation uncertainty was evaluated on both sample block and the ecoregion scales. In this study, values of selected output variables were written to a set of output files after each model execution, and then aggregated at four spatial levels: pixel (60 m × 60 m) → land use category → sample block (10 km × 10 km) → ecoregion.

### Tillage management data and actual tillage management scenario

Tillage management data were collected from the CTIC [24] for areas of annual CT, RT and conservational tillage practices (including mulch, ridge, and NT) and 8 crop types at a county scale for the period from 1989 to 1998. The areas for each combination of cropping system and tillage management were documented for individual counties at a two-year interval. For each combination of county, year, and crop type, the area extents were determined for the three tillage options.

**Figure 6:**
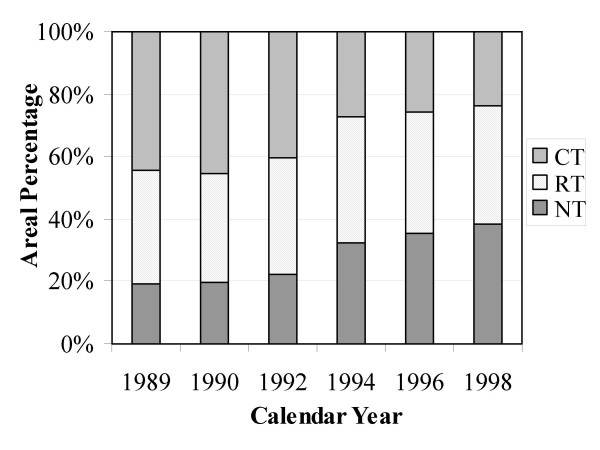
Areal percentage of each type of tillage management for the planted area of the Northwest Great Plains ecoregion (CT, conventional tillage; NT, no-till; RT, reduced-tillage).

The actual tillage management (ATM) information for model simulations was based on the CTIC data with an assumption that all planted area was managed with CT in 1972 and then converted to NT and RT until 1989 at a pace similar to that estimated from the CTIC data for the period between 1989 and 1998. The areas under ATM for all cropped lands are presented in Figure 6. The probability for each tillage management option for the years after 1998 was assumed to be the same as that in 1998. Tillage information for each crop was read in from the CTIC dataset to determine the three proportions of the total planted area for CT, RT, and NT. For a certain type of crop (e.g., corn), the fraction of each tillage type in 1992 was assigned as 0.5, 0.3, and 0.2 to planted areas managed with CT, RT, and NT, respectively.

### CT and NT scenarios for model simulations

To evaluate the effects of different tillage options on SOC dynamics across the ecoregion, we defined two extreme tillage scenarios for model simulations: (1) complete CT, which assumed that all planted areas were managed with conventional tillage since 1972, and (2) complete NT, in which all planted areas were assumed to be managed with no-till since 1972.

## List of abbreviations used

ATM – actual tillage management

CT – conventional tillage

GEMS – General Ensemble biogeochemical Modeling System

NT – no-till

RT – reduced tillage

SOC – soil organic carbon

## Competing interests

The author(s) declare that they have no competing interests.

## Authors' contributions

ZT designed this study, prepared model input, analyzed results, and drafted the manuscript. SL supervised this research and did model debug as the developer of GEMS. ZL was in charge of programming for incorporating tillage management data into model simulations and model debug. TRL provided the time-series land use/land cover change trends data and comments on the manuscript formulation. All authors read and approved the final manuscript.
